# Anesthesia and developing brain: What have we learned from recent studies

**DOI:** 10.3389/fnmol.2022.1017578

**Published:** 2022-11-21

**Authors:** Yixuan Niu, Jia Yan, Hong Jiang

**Affiliations:** Department of Anesthesiology, Shanghai Ninth People’s Hospital, Shanghai Jiao Tong University School of Medicine, Shanghai, China

**Keywords:** general anesthesia, neurotoxicity, neurodevelopmental impairment, cognitive dysfunction, developing brain

## Abstract

Anesthesia is unavoidable in surgical procedures. However, whether the general anesthetics are neurotoxic to immature brains remains undefined. Neurodevelopmental impairment induced by anesthesia has been a critical health issue and topic of concern. This review summarizes recent progress made in clinical and preclinical studies to provide useful suggestions and potential therapeutic targets for the protection of the immature brain. On the one hand, clinical researchers continue the debate about the effect of single and multiple exposures to anesthesia on developing brains. On the other hand, preclinical researchers focus on exploring the mechanisms of neurotoxic effects of general anesthesia on immature brains and seeking novel solutions. Rodent models have always been used in preclinical studies, but it is still unclear whether the mechanisms observed in rodent models have clinical relevance. Compared with these models, non-human primates (NHPs) are more genetically similar to humans. However, few research institutions in this area can afford to use NHP models in their studies. One way to address both problems is by combining single-cell sequencing technologies to screen differential gene expression in NHPs and perform *in vivo* validation in rodents. The mechanism of anesthesia-induced neurotoxicity still requires further elucidation in primates.

## Introduction

Anesthesia is unavoidable in surgical procedures, which often causes concerns for the parents of children who are scheduled to undergo surgery (Vutskits and Xie, 2016).General anesthetics act primarily by blocking N-Methyl-D-Aspartate (NMDA) recepters and/or activating gamma-aminobutyric acid (GABA) receptors. Each year, an increasing number of children receive surgical intervention under general anesthesia. Nervous system development is at its peak and is very sensitive to anesthesia from mid-gestation to the postnatal year 2 or 3 (Yan and Jiang, 2014). Exposure to anesthesia during this period may have neurotoxic effects on developing brains (Sun, 2010). In 2016, the United States Food and Drug Administration (FDA) warned that prolonged or repeated exposures to general anesthetics in late pregnancy or before 3 years old may have effects on the developing brain (Davidson and Sun, 2018). Therefore, investigations of the neurotoxic effects of general anesthesia on immature brains may have important clinical implications.

Over the years, numerous clinical and preclinical studies have been conducted to improve the safety of general anesthesia exposure and find potential novel targets to protect the immature brain (Warner et al., 2018; Yan et al., 2020; Warner et al., 2021; Zhang et al., 2022). Therefore, the effect of general anesthetics on the developing brain remains a source of intense debate.

## Clinical evidence of anesthesia-induced neurodevelopmental outcomes: Effect of single and multiple exposures to anesthesia

The warnings issued by the FDA have been the driving force behind numerous clinical and preclinical studies. Due to ethical and technical restrictions, it is hard to perform prospective, randomized studies on human fetuses and neonates without confounding factors. Although many animal studies have shown the neurotoxic effects of general anesthetics on the immature brain, there are few studies on what effect general anesthetics have on human fetuses and neonates (Andropoulos, 2018). Consequently, the effects of general anesthetics on human fetuses can only be studied in animal models. However, the outcomes of preclinical studies in animal models can be validated in clinical studies.

Several clinical studies have been conducted to further explore the side effects general anesthetics have on immature brains, including three well-known clinical studies, which are the General Anesthesia vs. Spinal Anesthesia (GAS) study, the Pediatric Anesthesia, and Neurodevelopment Assessment (PANDA) study, and the Mayo Anesthesia Safety in Kids (MASK) study (Davidson et al., 2016; Sun et al., 2016; Davidson and Sun, 2018). The GAS studies were reported in 2016 and 2019, which indicated that there was no evidence showed that single exposure to sevoflurane for less than 1 h in early infancy, compared with awake-regional anesthesia, increased the risk of changing neurodevelopmental outcomes at 2 years of age and 5 years of age (Davidson et al., 2016; McCann et al., 2019). Another critical study called “the PANDA study” used 105 sibling pairs to demonstrate that there were no statistically significant differences in intelligence quotient (IQ) scores in later childhood between children who used to receive single anesthesia exposure under 36 months old and siblings with no anesthesia exposure (Sun et al., 2016). However, the idea that single and multiple exposures are relatively safe for children was proved by another important study called the MASK study. Children under 3 years old were recruited in this study to demonstrate that single or multiple exposures to general anesthesia were not associated with deficits in the primary outcome of general intelligence (Warner et al., 2018). These three studies together put forth the idea that no evidence supports single or multiple anesthesia exposures in early infancy reduce IQ scores in later childhood.

Additionally, these three clinical studies found that multiple exposures to anesthesia might lead to behavioral and executive function deficits. The results of the secondary analysis of this study published in 2021 proposed a new idea that multiple, but not single, exposures might cause adverse effects on specific neuropsychological domains, such as processing speed, fine motor abilities, motor skills, and visual-motor integration (Warner et al., 2018, 2021). Compared with the two studies above, it was a significant advancement that the MASK study moved our attention from “single exposure” to “multiple exposures.”

In addition to the three well-known clinical studies above, other remarkable studies were recently published. A cohort study in Avon was recently published in 2020, in which 13,433 children grouped by none, single, and multiple exposures to anesthesia were recruited, and the results indicated that multiple exposures might cause adverse effects on motor ability and social, linguistic ability (Walkden et al., 2020). Recently, a national population-based cohort study compared 11,457 children who received general anesthesia under 2 years old with 22,914 children without exposure to anesthesia and demonstrated that early exposure (before 2 years old) to anesthesia may increase the risk of developmental delay (DD; Feng et al., 2020). Furthermore, the risk was associated with the frequency and duration of anesthesia.

Recently, an observational study has revealed that children under the age of 5 years with a single exposure to surgery and anesthesia were 37% more likely to need attention-deficit hyperactivity disorder (ADHD) medications than unexposed children, which means even single exposure to anesthesia might be associated with deficits in behavior and executive function (Ing et al., 2020). In contrast, the results of secondary analysis of the MASK study indicated that this analysis did not find evidence that single exposure to general anesthesia under 3 years of age would lead to ADHD and learning disabilities (LD) later in their lives (Warner et al., 2021). Consequently, the two studies have come to inconsistent conclusions. Compared with the MASK study, there are some deficiencies in the observational study conducted by Ing et al. (2020). On the one hand, in addition to ADHD medications, differences were also seen in the use of other drugs, such as amoxicillin, azithromycin, and diphenhydramine (Ing et al., 2020). On the other hand, ADHD medication use depends on the presence of adequate concern by the parents to some extent (Ing et al., 2020). As a result, the conclusion should be treated with caution.

**Figure 1:**
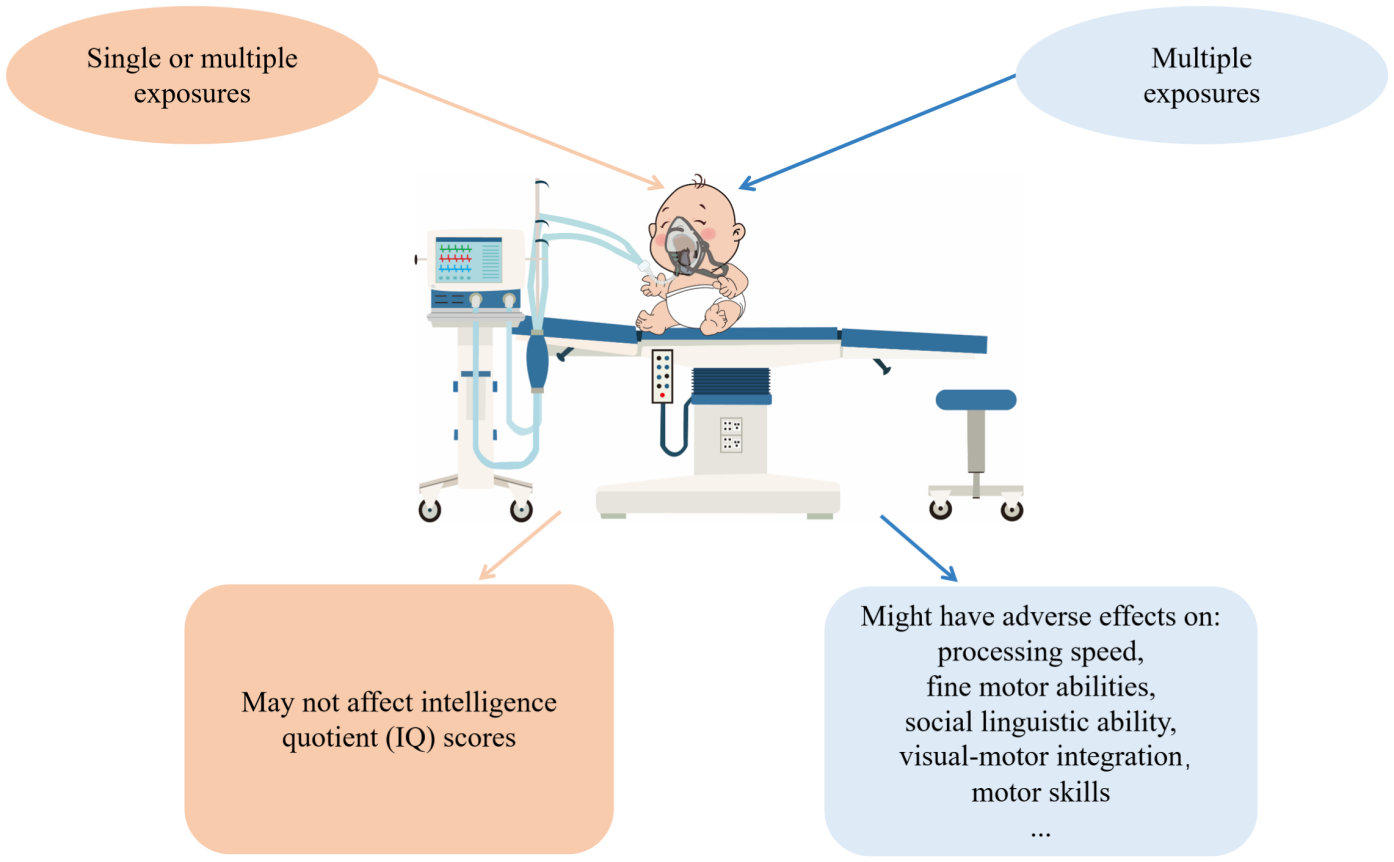
The effects general anesthetics have on human fetuses. According to clinical evidence of anesthesia-induced neurodevelopmental outcomes, no evidence supports that single or multiple exposures to general anesthesia reduce IQ scores in later childhood. In contrast, multiple exposures may adversely affect processing speed, fine motor abilities, social, linguistic ability, visual-motor integration, and motor skills.

Finally, the results of the above studies have provided strong evidence that single or multiple exposures to general anesthesia may not lead to reduced IQ scores in later childhood, while receiving multiple exposures to anesthesia might increase the risk of neurodevelopmental impairment and long-term cognitive dysfunction (Figure 1). Anesthesiologists may communicate with patients and postpone non-urgent procedures to prevent exposing children to anesthesia multiple times during their vulnerable period unless the operation is urgently needed. However, receiving multiple anesthesias means receiving multiple surgery operations as well. The surgeries themself may likely be associated with the risk of adverse neurodevelopmental outcomes (Ing et al., 2020). In addition, children with congenital disorders are more likely to undergo multiple surgeries under 3 years old. Injuries come with operations, and congenital disorders might further bias the results.

However, clinical research cannot address or resolve all the problems of anesthesia-induced neurotoxicity. Only preclinical studies could identify which mechanisms are implicated in neurotoxicity (Salaün et al., 2021). Therefore, conducting preclinical studies to explore mechanisms of anesthesia-induced neurodevelopmental impairment is essential.

## Preclinical evidence: Exploring mechanisms of anesthesia-induced neurodevelopmental impairment

Preclinical research will always be a major actor in research on the toxicity of general anesthesia. Tremendous attention has been paid to anesthesia-induced neurotoxicity in the immature brain. Parents’ anxiety is almost inevitable when referring to the unsure risk of anesthesia to children. Unnecessary parental anxiety is one of the controversies regarding anesthesia-related neurotoxicity (Barnes, 2020). Commonly used anesthetic agents are thought to exert their effect via NMDA and GABA receptors (Rappaport et al., 2011). According to studies conducted by the National Center for Toxicology Research (NCTR) of the Food and Drug Administration (FDA), exposure to ketamine, which is the prototypical NMDA-receptor antagonist, might resulted in increased neuronal cell death in NHPs (Slikker et al., 2007). Another study conducted by Brambrink et al. (2010) confirmed that Isoflurane, which is predominantly a GABAergic agent, may cause a large increase in neuronal apoptosis in several brain regions (Brambrink et al., 2010). When children are exposed to general anesthetics, the mechanism by which general anesthetics lead to neurodevelopmental impairment and long-term cognitive dysfunction is complex. Therefore, efforts made by researchers on this topic are still warranted. Fortunately, scientific research output in recent years has provided us with more opportunities to explore the mechanism of anesthesia-induced neurotoxicity (Ing et al., 2022).

### Is apoptosis a defender or a natural-born killer

Apoptosis is a kind of programmed cell death that occurs in many cell types due to various circumstances and aging (Brambrink et al., 2010). Exposures of infant mice, rats, and rhesus monkeys to general anesthetics might lead to widespread apoptotic death of neurons in the developing brain (Brambrink et al., 2010; Raper et al., 2018). A recent study found that exposing 6-week-old rhesus monkeys to sevoflurane three times for 4 h each time appeared to be at high risk of delayed-onset mild visual memory deficits by age 1 and anxiety behavior at the age of 2 (Raper et al., 2018). Although the study did not explain the mechanism of the changes, it did raise the possibility that this kind of change was likely the consequence of neuronal and glial cell apoptosis (Alvarado et al., 2017; Raper et al., 2018). Notably, a 2-year-old rhesus monkey is similar to a 6-year-old human (Raper et al., 2018). As is well known, disturbances in emotional behavior at this age might have a long-term effect on social abilities and academic achievement.

According to recent studies, intranasal use of insulin appears to be a feasible approach to protect the vulnerable immature brain from anesthesia-induced apoptosis. Intranasal use of insulin can bypass the blood–brain barrier to deliver insulin to the hippocampus and other cerebral regions and bind to their receptors (Li et al., 2019). By performing the animal experiment, the studies reported that intranasal use of insulin might activate the mechanistic target of rapamycin complex 1 (mTOR-1) to promote mitochondrial functions and rescue apoptosis (Li et al., 2019; Dai et al., 2020). Mitochondrial dysfunction plays an important role in anesthesia-induced neurotoxicity (Boscolo et al., 2013). Exposure to general anesthetics might result in an excessive production of reactive oxygen species (ROS), mitochondrial fragmentation and cytochrome c release from mitochondria into the cytosol, which subsequently cause caspases-3 activation and apoptosis (Jevtovic-Todorovic et al., 2012; Boscolo et al., 2013; Roque et al., 2021). mTOR-1 is an essential intracellular effector of the insulin receptor and many other metabolic processes (Liu and Sabatini, 2020; Roque et al., 2021). By activating mTOR-1, intranasal insulin may promote mitochondrial functions and contribute to resistance of cells to anesthesia-induced cellular stress (Roque et al., 2021).

In addition, the extent of apoptosis is probably associated with age and gender differences. Exposing P7 rats to isoflurane 4 h leads to decreased recognition memory in male but not female rat (Lee et al., 2014). According to a recent study, postnatal day 4 (p4) female rats were more vulnerable to anesthetic neurotoxicity and memory deficits than postnatal day 7 (P7) female rats when exposed to isoflurane 1 minimum alveolar concentration for 4 h (Sasaki Russell et al., 2019). The potential mechanism might be associated with the expression of chloride transporters K + -Cl– co-transporter 2 (KCC2) and Na + -K + -Cl– co-transporter 1 (NKCC1), which is vital to normal brain development. This study showed that by P7, the female cortex expressed more KCC2 and less NKCC1 than in males. The ratio of NKCC1/KCC2 expression in cerebral cortex was higher in P4 females than in P7 females, but comparable to data for P7 male rat. Compared to P7 female rats, P4 female rats showed a significant increase in the level of apoptosis in the hippocampus, medial dorsal thalamus, and lateral dorsal thalamus, which impaired spatial reference memory and associative recognition memory.

Gender differences can also affect the immature brain *via* other molecular mechanisms. In the above study, researchers observed that testosterone reduces KCC2 expression, leading to developmental retardation of brains (Sasaki Russell et al., 2019). Other studies have also demonstrated that testosterone may be neurotoxic to immature brains. By extending the vulnerable period of anesthesia, testosterone also increases the extent of sex-specific neurodevelopmental impairment (Sasaki Russell et al., 2021). However, testosterone might have dual effects on neurodevelopment. Recent studies have proposed that testosterone inhibits the interaction between microtubule-associated protein tau and glycogen synthase kinase-3β (GSK3β) to attenuate tau phosphorylation (Yang et al., 2021; Jevtovic-Todorovic, 2022). Increased tau phosphorylation is associated with altering dendritic spines’ plasticity (Pallas-Bazarra et al., 2016), which is related to long-term cognitive dysfunction.

Previous studies have reported that a long duration of 3% sevoflurane impaired cognitive functions through apoptosis. In comparison, a single and short duration of 2% sevoflurane (clinical concentration) might lead to cognitive dysfunction *via* underlying mechanisms other than apoptosis (Lu et al., 2016). However, its specific mechanism is still unclear, and added research is warranted.

### Neuroinflammation is one of the primary culprits

Neuroinflammation is the most commonly mentioned mechanism when talking about anesthesia-induced neurodevelopmental impairment. Microglia can be polarized into the classical activation (M1), which is harmful to neurons, and the alternative activation (M2), which is helpful for tissue repair (Boche et al., 2013). A well-balanced proportion of M1 and M2 is essential to maintaining the normal functions of nervous cell (Huang et al., 2021). However, sevoflurane might enhance M1 polarization and simultaneously suppress M2 activation (Pei et al., 2017). According to Zhang et al., sevoflurane and isoflurane might induce neuroinflammation and cognitive impairment by increasing the level of interleukin-6 (IL-6) *via* nuclear factor-kappa B (NF-κB) signaling, which is associated with M1 activation (Zhang et al., 2013).

As a part of the mitogen-activated protein kinase (MAPK) signaling pathway, the JNK signal transduction pathway is associated with the secretion of IL-6. A recent study demonstrated the functional role of lncRNA Riken in attenuating sevoflurane-induced neuroinflammation and also revealed the underlying miRNA-101a-associated molecular mechanisms (Hou et al., 2021). One function of lncRNA is to stick to miRNA like a sponge, thus inhibiting miRNA from combining to 3′UTR of target mRNA——downregulating the ability of miRNA *via* the mechanism of ceRNA (Tay et al., 2014). By inhibiting miRNA-101a from targeting MAPK phosphatase 1 (MKP-1), lncRNA Riken alleviated the sevoflurane-induced neurotoxic effects (Hou et al., 2021).

Pyroptosis is a novel inflammatory form of programmed cell death defined as gasdermin-mediated programmed necrosis (Shi et al., 2015, 2017). A recent study has found that exposing P6 rats to 3% sevoflurane 2 h daily for three consecutive days can activate NF-κB signaling to release pyroptosis-related proteins such as caspase-1, caspase-11, and nod-like receptor pyrin domain-containing 3 (NLRP3) (Dai et al., 2021). Gasdermin D (GSDMD) is the substrate for active caspase-1 and caspase-11. Additionally, activated inflammatory caspases might cleave Gasdermin D (GSDMD) protein into N-terminal and C-terminal fragments (Shi et al., 2017). One function of the N-terminal fragment is to form membrane nanoscopic pores, leading to cell swelling and proinflammatory cytokine release. Importantly, activated NF-κB is an upstream activation mechanism. Therefore, inhibiting NF-κB signaling may be feasible to rescue sevoflurane-induced pyroptosis and inflammation and attenuate cognitive dysfunction in adulthood (Dai et al., 2021).

A series of animal experiments have suggested that NF-κB is an essential regulator of many pathways of neuroinflammation (Zhang, Zhang et al., 2013, Shi et al., 2017, Dai et al., 2021, Huang et al., 2021). By inhibiting NF-κB signaling, both canonical (caspase-1 indicated) and non-canonical (caspase-11 indicated) inflammatory responses can be blocked (Dai et al., 2021). Furthermore, it provides a potential therapeutic target to protect immature brains from anesthesia-induced neurotoxicity and developmental impairment.

### Oxidative stress and antioxidant imbalance

A fine balance between the presence of ROS and antioxidants is crucial for the proper normal functioning of the cell (Senoner et al., 2021). Exposure to general anesthetics during the early stages of the central nervous system can easily disturb this balance and lead to neurotoxicity (Jevtovic-Todorovic et al., 2013). However, surgical procedures are also associated with an increase in oxidative stress (Arsalani-Zadeh et al., 2011). To prevent aggravation of brain damage, anesthesiologists may choose general anesthetics after much deliberation. Specifically, sevoflurane and desflurane might induce oxidative stress, while propofol seems to perform well in reducing oxidative stress (Senoner et al., 2021).

Usually, doctors tend to focus only on how hypoxia disrupts normal functions of immature brains, which may not always be correct. Normal brain functions are based on maintaining the partial pressure of brain tissue oxygen within a relatively narrow range. Damage to the immature brain has been related to hypoxia, while hyperoxia-induced neurotoxicity should not be underestimated. Administration of general anesthetics might increase the level of ROS by elevating partial pressure of brain tissue oxygen of neonates, then disrupt cognitive functions in immature brains (Aksenov et al., 2018). Additionally, there was relevant morphological and functional damage in Purkinje cells in the developing cerebellum caused by neonatal hyperoxia (Scheuer et al., 2018).

Well-balanced ROS and antioxidants is crucial to the proper development of the central nervous system. Hypoxia might lead to injured brain tissues, while the effects of hyperoxia should also be considered. To prevent aggravation of brain damage, anesthesiologists may choose general anesthetics after much deliberation. In addition, clinical use of near-infrared spectroscopy (NIRS)-based cerebral oximetry provides conditions for monitoring brain tissue oxygen (Aksenov et al., 2018).

### Why synaptic plasticity alters

Synaptogenesis in the human neocortex occurs from the third trimester to the postnatal 2 or 3 years (Yan and Jiang, 2014). A series of animal experiments have suggested that early exposure to general anesthetics might affect synaptic plasticity, and abnormal synaptic functions are associated with cognitive dysfunction (Bello-Medina et al., 2016; Pallas-Bazarra et al., 2016; Li et al., 2019; Sasaki Russell et al., 2019; Schaefer et al., 2020; Wan et al., 2021). Additionally, the postsynaptic structural protein PSD-95 can be a synaptic plasticity marker (Kim and Sheng, 2004). When PSD-95 decreases, postsynaptic structure changes might be the underlying mechanism that leads to this effect. A recent study has described a significant decrease in the number of PSD-95 in the cerebral cortex and the hippocampus after exposing P7 neonatal mice to 3% isoflurane for 3 h per day for 3 consecutive days (Li et al., 2019). In this article, intranasal use of insulin before exposure to isoflurane may regulate synaptic density, thus protecting the cognitive functions of the immature brain.

Dendritic spines include different types of spines, such as thin spines, defined as “learning spines,” and mushroom spines, defined as “memory spines.” Dendritic spines receive excitatory input, and their morphology is strongly related to learning and memory abilities (Bourne and Harris, 2007). Long-term potentiation associated with learning might convert thin spines to mushroom spines, whereas anesthesia-induced morphological shift from thin spines to mushroom spines may lead to learning dysfunctions. Previous studies have shown that exposure to anesthesia might increase tau’s phosphorylation level, which may decrease the proportion of thin spines and reduce learning abilities (Bello-Medina et al., 2016; Pallas-Bazarra et al., 2016). However, stable mushroom spines, defined as “memory spines,” can also disrupt memory abilities when reduced. The PDZ domain of PSD-95 is a molecular target for inhaled anesthetics (Fang et al., 2003). Interaction of the PDZ domain of PSD-95 and N-methyl-d-aspartate (NMDA) receptor NR2 subunits helps promote synaptogenesis. Studies by Schaefer and colleagues suggest that exposure of P7 mice to 1.5% isoflurane for 4 h or PSD-95 PDZ2WT peptide decreases the efficiency of neuronal NO synthase (nNOS) by inhibiting protein–protein interaction between PSD-95 PDZ, NMDA NR2, and nNOS. These effects, therefore, lead to reduced thin spines, impairment of long-term potentiation induction, and a decrease in mushroom spines (Schaefer et al., 2019, 2020). Learning and memory that involve the amygdala and hippocampus were disrupted, while the administration of nitric oxide donor can prevent this disruption. This indicated that the NMDA NR2/PSD-95 PDZ2/nNOS pathway played an influential role in the cognitive functions resulting from altered synaptic plasticity (Schaefer et al., 2019).

Propofol is popular among intravenous general anesthetics (Kotani et al., 2008). According to a recent study, early exposure to this general anesthetic is associated with adult cognitive dysfunction (Wan et al., 2021). Exposing P7 rats to 60 mg/kg propofol for three consecutive days might lead to marked impairment of hippocampal synaptic plasticity and cognitive dysfunction in adult rats (Wan et al., 2021). However, the underlying mechanism of propofol anesthesia impairs hippocampal synaptic plasticity still needs further investigation.

Other studies revealed that sex hormones might contribute to synaptic changes. As discussed above, testosterone reduces KCC2 expression, leading to neurodevelopmental retardation of brains (Sasaki Russell et al., 2019). The underlying mechanism might be that reduced KCC2 and NKCC1 ratios lead to the relatively slow maturation of neural circuitry. As a result, gender differences should be considered in real applications.

Additionally, exposure to general anesthetics may disrupt normal mitochondrial function. Mitochondrial unfolded protein response (UPRmt) helps upregulate mitochondrial function and frequency of miniature excitatory postsynaptic currents when exposed to general anesthetics (Lee et al., 2021). Therefore, activation of UPRmt may be the underlying mechanism of increased excitatory synaptic transmission (Lee et al., 2021). Notably, attention to anesthesia-induced synaptic changes might help provide a potential therapeutic target.

### Autophagy may be a double-edged sword

Autophagy is a process of natural degradation that can help maintain metabolic stability (Jevtovic-Todorovic et al., 2012). Microtubule-associated protein one light chain 3 (LC3) is a key protein involved in autophagy, which is commonly used as an autophagy marker (Huang and Liu, 2015). LC3 converts from soluble form (LC3-I) to autophagy-associated form (LC3-II) during the elongation progress of autophagy (Fernández and López-Otín, 2015). Exposing P6, P7, and P8 rats to 3% sevoflurane for 2 h 1 day might lead to cognitive dysfunction in neonatal mice by increasing the level of LC3-II, LC3-II, and LC3-I ratio, Beclin-1 and decreasing the level of p62 in the hippocampus (Xu et al., 2018; Wang et al., 2019). Autophagy is initiated by a combination of class III phosphoinositide 3-kinases (PI3Ks) and Beclin-1 (an autophagy-involved protein) (Su et al., 2015). Previous studies have confirmed that the PI3K-AKT–mTOR pathway is involved in the activation of autophagy. According to Wang et al., sevoflurane-induced cognitive impairment in young mice might be restored by administration of 3-methyladenine (3-MA), which blocks autophagic vesicle formation *via* inhibition of phosphoinositide 3-kinase, thus blocking the formation of the autophagic vesicle (Wang et al., 2019).

However, autophagy might still play a role in neuroprotection. A recent study demonstrated that roscovitine (Rosc) administration inhibited the activation of Cyclin-dependent kinase 5 (CD5), which can upregulate SIRT1-induced autophagy. SIRT1-induced autophagy might reverse sevoflurane-induced neurodevelopmental impairment and cognitive dysfunction (Yang et al., 2020). However, the molecular mechanism of SIRT1-regulated autophagy is unclear. Therefore, the role of autophagy in cognitive dysfunction still needs further investigation.

### Myelination deficit is one of the key mechanisms of anesthesia-induced cognitive dysfunction

Proper myelin formation is essential to neurocognitive function. In the central nervous system, oligodendrocytes (OLs) are a particularly specialized type of glial cell which provide fundamental support to neuronal activity by producing the myelin sheath (van Tilborg et al., 2016; Wu Z. et al., 2020). However, iron deficiency induced by sevoflurane might inhibit the proliferation of oligodendrocytes, thereby leading to disrupted myelin development (Wu Z. et al., 2020; Zuo et al., 2020). Maternal exposure to 2% sevoflurane for 6 h on gestation stage day 14 might cause cognitive impairment in postnatal day 35 mice by decreasing iron and inhibiting myelin formation in the hippocampus and cerebral cortex (Zuo et al., 2020). Notably, iron plays an essential role in immature brain development (Golonka et al., 2019). Therefore, it is necessary to give iron therapy to a neonatal whose mother used to accept anesthesia during pregnancy.

Additionally, disrupted folate metabolism is associated with myelination deficits as well. A study performed by Zhang and colleagues showed that sevoflurane decreased TYMS (an essential gene for the folate-dependent enzyme) and ERMN (a myelination-related gene) levels in both rhesus macaques and mice. Additionally, the expression of TYMS and ERMN is strongly related to folate metabolism (Zhang et al., 2019). Exposing P7, P21, and P35 rhesus macaques and P6, P7, and P8 mice to 2.5–3% sevoflurane 4 h might reduce the expression of TYMS and folate, which affect DNA methylation, thereby further affecting the expression of ERMN (Zhang et al., 2019). Downregulated ERMN might be one of the potential mechanisms of myelination deficits and hippocampus-dependent spatial learning and memory dysfunction in young animals and children. Collectively, administration of folic acid and restoration of ERMN signaling can help rescue the myelination defects and attenuate anesthesia-induced cognitive dysfunction.

Myelination deficit is one of the key mechanisms of anesthesia-induced cognitive dysfunction. Iron therapy, folic acid supplementation, and ERMN are well-established therapeutic regimens for patients. These studies provide more targets for protecting immature brains in clinical works by discovering the underlying mechanisms of myelination deficits.

### Inhibition of neural differentiation and new ideas for future work

Dual specificity protein phosphatase 4 (Dusp4) plays a crucial role in neural differentiation, which can mediate the differentiation from human embryonic stem cells (hESCs) to neural progenitor cells (NPCs) (Yan et al., 2020). According to a related study (Yan et al., 2020), exposing P7, P21, and P35 rhesus macaque and P6, P7, and P8 mice to 2.5–3% sevoflurane 4 h might inhibit neural differentiation of non-human primates (NHPs) by downregulating the expression level of Dusp4. However, sevoflurane treatment-caused Dusp4 downregulation was specifically in NHPs rather than rodents. Notably, there are many differences between rodents’ and primates’ developmental specificity and the nervous system (Xue et al., 2013). Furthermore, the mechanism of neurotoxicity varies in different species. NHPs are 98% genetically similar to humans compared with rodents and can, in many instances, more accurately predict how pathological conditions arise in the human body (Wang et al., 2017). In other words, using NHPs may help explore a more reliable mechanism that still needs further investigation.

According to the above study, we concluded that downregulated level of Dusp4 in the prefrontal cortex appeared in NHPs but not in rodents. This study provides us with a potential therapeutic target while reminding us that the mechanism of neurotoxicity may not be the same between NHPs and rodents. Although few research institutions in this area can afford to use NHP models, the article provides new ideas for future work, such as combining single-cell sequencing technologies to screen differential gene expression in NHPs and carry out *in vivo* validations in rodents. The combination of NHP and rodent models may further elucidate our understanding in human subjects.

### Ferroptosis is one of the novel mechanisms of anesthesia-induced neurotoxicity in developing brain

Ferroptosis is another novel kind of programmed cell death characterized by iron-dependent lipid peroxides accumulation (Angeli et al., 2017). Glutathione peroxidase 4 (GPX4) can prevent the accumulation of lipid peroxides and ROS (Angeli et al., 2017). When exposed to isoflurane, the activity of GPX4 is inhibited, and accumulated lipid peroxides and ROS might lead to disruption in mitochondrial membrane potential and cell death (ferroptosis) (Xia et al., 2018). In addition, exposure to isoflurane might increase the activity of the mitochondrial electron transport chain (Liu et al., 2021). These processes can be attenuated by administering selective ferroptosis inhibitor ferrostatin-1 and mitochondria activator dimethyl fumarate (Xia et al., 2018; Liu et al., 2021).

Meanwhile, exposure to sevoflurane might also lead to iron-induced neurotoxicity in the hippocampus and cognitive dysfunction by upregulating NMDA receptors and enhancing divalent metal transporter 1 (DMT1)-mediated iron upregulation and iron release from lysosome (Wu J. et al., 2020). The damage might be attenuated by chelating neurotoxic iron. These findings of ferroptosis encouraged more investigators to explore underlying mechanisms of anesthesia-induced neurodevelopmental impairment and cognitive dysfunction, thus providing more potential therapeutic targets.

### Clinical concentration might lead to dysregulation of gut microbiota

The dysregulation of the gut microbiota might also influence the immature brain. A recent study has shown that the clinical concentration of sevoflurane did affect the gut microbiota. Exposing 6 to 8-week-old mice to 2.21% sevoflurane for 4 h might reduce the microbiome’s variety in neonatal mice (Han et al., 2021). Microbiota profiling analysis showed that some differentially abundant taxa are related to memory. Studies of gut microbiota dysregulation might provide a therapeutic strategy with novel insights (Liu et al., 2021).

## What have we learned from recent studies

On the one hand, most clinical studies have paid attention to the dose, timing, and frequency of exposure. According to the above clinical studies, single or multiple exposure might not reduce IQ scores. In contrast, multiple exposures may adversely affect processing speed, fine motor abilities, social, linguistic ability, visual-motor integration, and motor skills. Therefore, parents do not need to worry excessively about single and short exposure. In addition, future clinical studies should give greater attention to prospective, multicenter, and long-term follow-up clinical studies.

**Figure 2:**
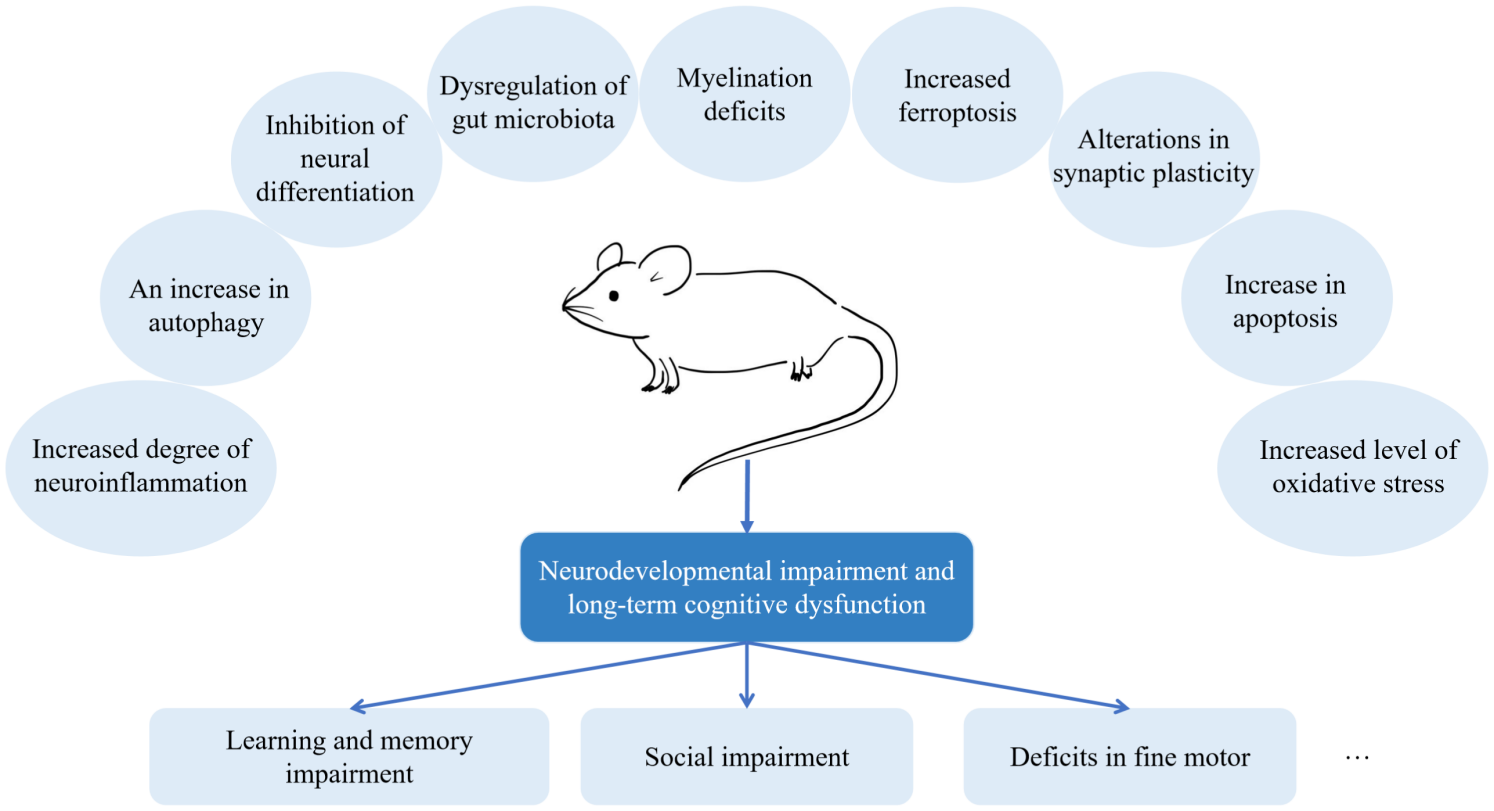
Mechanisms of anesthesia-induced neurodevelopmental impairment. It is challenging to recognize which is the most important mechanism *in vivo*. According to recent preclinical studies, different anesthetic agents might induce neurotoxicity through various mechanisms.

On the other hand, preclinical studies resolve the problem of how general anesthetic agents induce neurotoxicity, a challenging area of clinical studies. There has been much discussion about the potential mechanisms of general anesthesia-induced neurotoxicity (Figure 2). However, it is difficult to recognize which mechanism is the most important *in vivo*. Different anesthetic agents might induce neurotoxicity through various mechanisms. In addition, the translational value of preclinical studies is limited by multiple factors. First, although most preclinical studies are conducted under general anesthesia, they are not stimulated by surgeries. Clinically, this is virtually absent. Children and pregnant women receive general anesthesia for surgeries rather than only exposing to anesthesia. Second, due to a species difference, the results found in rodent models may not fully explain the mechanisms in humans. Therefore, we need to improve the quality of experiments and look for animal models that best suit clinical situations.

Rodent models have always been used for exploratory studies. However, it is difficult to translate preclinical outcomes into clinical practice because it is still unclear whether the mechanisms observed in rodent models have clinical relevance (Salaün et al., 2021). Compared with rodents, NHPs are more genetically similar to humans (Wang et al., 2017). Nevertheless, due to the limited number and the high costs of rhesus macaques, NHPs studies are limited. Few research institutions in this area can afford to use NHP models in their studies. One way to address both problems is by combining single-cell sequencing technologies to screen differential gene expression in NHPs and perform *in vivo* validation in rodents. Notably, studies have suggested that this trend will continue in the future (Zhang et al., 2019; Yan et al., 2020).

## Conclusion

**Figure 3:**
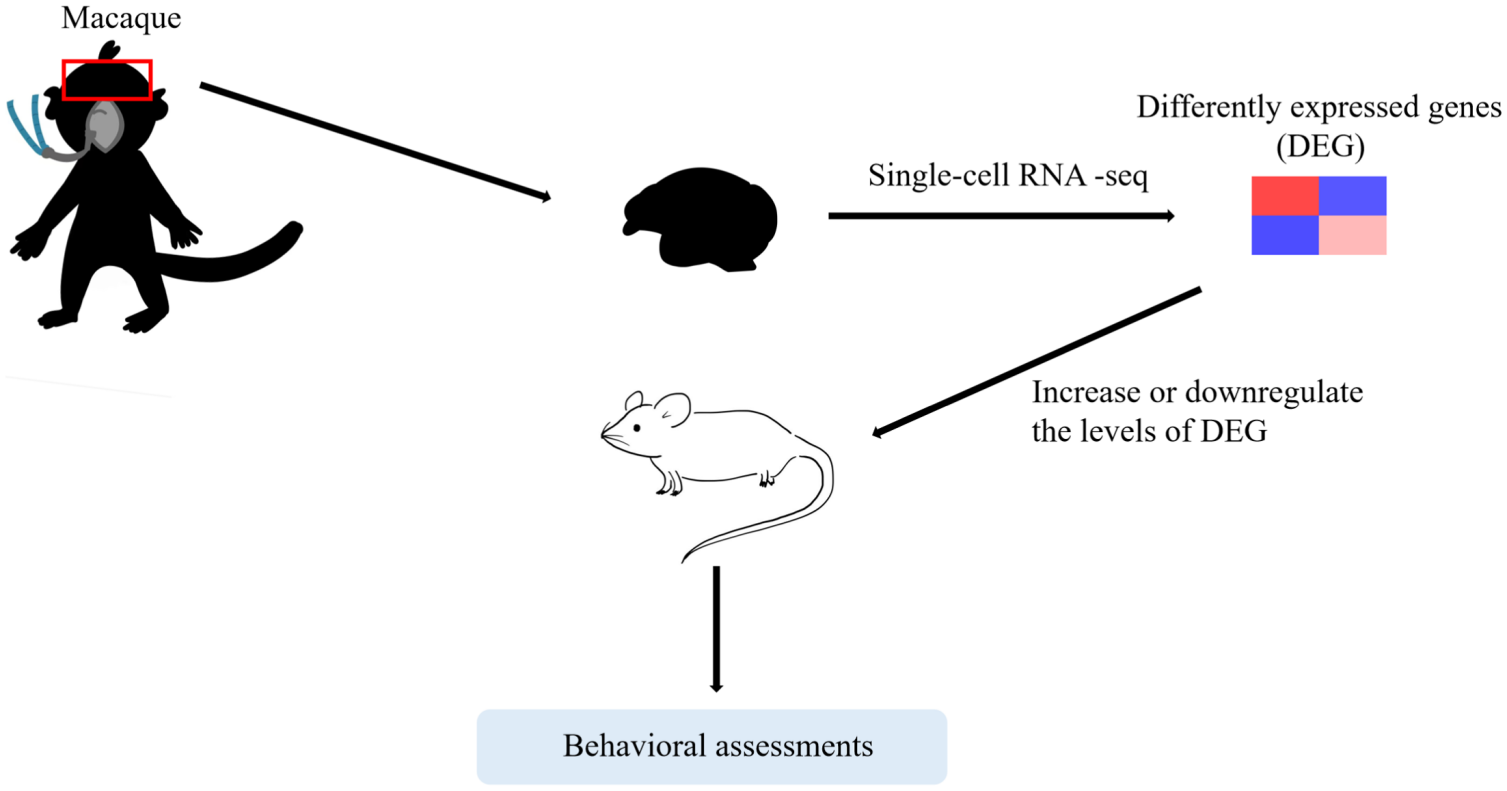
A trend that may continue in the future. One way to address both problems is by combining single-cell sequencing technologies to screen differential gene expression in NHPs and carry out *in vivo* validations in rodents.

Herein, we have reviewed the current clinical and preclinical studies concerning effects on immature brains. Clinically, there is no need for parents to excessively worry about the single and short exposure to general anesthesia. Additionally, we recommend anesthesiologists communicate with patients and postpone non-urgent procedures to prevent children from exposing to anesthesia multiple times during their vulnerable period unless the operation is urgent. Meanwhile, exploring mechanisms provides us with more therapy strategies with novel insights. Currently, limitations still exist in preclinical studies, specifically in the use of their applicable models. These limitations suggest directions for future improvements, such as combining single-cell sequencing technologies to screen differential gene expression in NHPs and carry out *in vivo* validations in rodents (Figure 3).

## Author contributions

JY and HJ designed and revised the manuscript. YN wrote the draft. All authors contributed to the article and approved the submitted version.

## Funding

This work was supported by the National Natural Science Foundation of China (Grant No. 81870818), the Natural Science Foundation of Shanghai (Grant No. 22ZR1437200), and the scientific research fund of Shanghai Ninth People’s Hospital (Grant No. JYHX2021012).

## Conflict of interest

The authors declare that the review was conducted in the absence of any commercial or financial relationships that could be construed as a potential conflict of interest.

## Publisher’s note

All claims expressed in this article are solely those of the authors and do not necessarily represent those of their affiliated organizations, or those of the publisher, the editors and the reviewers. Any product that may be evaluated in this article, or claim that may be made by its manufacturer, is not guaranteed or endorsed by the publisher.
